# A pilot study of orthopaedic resident self-assessment using a milestones’ survey just prior to milestones implementation

**DOI:** 10.5116/ijme.5682.6dfd

**Published:** 2016-01-11

**Authors:** Kendall E. Bradley, Kathryn M. Andolsek

**Affiliations:** 1 Orthopaedic Surgery Residency Training Program, Duke University Hospital, Box 3000 DUMC, Durham, NC 27710, USA; 2 Community and Family Medicine, Duke University School of Medicine, Box 3648 DUMC, 201 Trent Drive, Durham, NC 27710, USA

**Keywords:** Postgraduate medical education, competency based education, self-assessment, graduate medical education, milestonesPostgraduate medical education, competency based education, self-assessment, graduate medical education, milestones

## Abstract

**Objectives:**

To pilot test if Orthopaedic Surgery residents could self-assess their performance using newly created milestones, as defined by the Accreditation Council on Graduate Medical Education.

**Methods:**

In June 2012, an email was sent to Program Directors and administrative coordinators of the154 accredited Orthopaedic Surgery Programs, asking them to send their residents a link to an online survey. The survey was adapted from the Orthopaedic Surgery Milestone Project. Completed surveys were aggregated in an anonymous, confidential database. SAS 9.3 was used to perform the analyses.

**Results:**

Responses from 71 residents were analyzed. First and second year residents indicated through self-assessment that they had substantially achieved Level 1 and Level 2 milestones. Third year residents reported they had substantially achieved 30/41, and fourth year residents, all Level 3 milestones. Fifth year, graduating residents, reported they had substantially achieved 17 Level 4 milestones, and were extremely close on another 15. No milestone was rated at Level 5, the maximum possible.  Earlier in training, Patient Care and Medical Knowledge milestones were rated lower than the milestones reflecting the other four competencies of Practice Based Learning and Improvement, Systems Based Practice, Professionalism, and Interpersonal Communication. The gap was closed by the fourth year.

**Conclusions:**

Residents were able to successfully self-assess using the 41 Orthopaedic Surgery milestones. Respondents’ rate improved proficiency over time. Graduating residents report they have substantially, or close to substantially, achieved all Level 4 milestones. Milestone self-assessment may be a useful tool as one component of a program’s overall performance assessment strategy.

## Introduction

The Accreditation Council for Graduate Medical Education (ACGME) is the accrediting body for the United States Graduate Medical Education system. It encompasses over 115,000 residents and fellows training in over nine thousand programs.^1^ Approximately 3 percent of all residents are in ACGME Orthopaedic Surgery programs.^2^

Since 1999, ACGME has championed competency-based education (CBE), identifying six core competencies expected of all physicians regardless of specialty.^3^ Orthopaedic Surgery identified the lack of “formal” delineated Orthopaedic-specific competencies as a key barrier in implementing CBE. Orthopaedic program directors and residents have recommended both clearer definitions and a greater focus on key surgical procedures. In 2008, twelve Orthopaedic educators from 9 programs recommended a series of changes in Orthopaedic education; prominent among these was a call for “technical and/or psychomotor skill benchmarks by year of residency.” ^4^^,^^5^

The newly created milestones may address the need for more specific targets within Orthopaedic residency training. In 2009, ACGME initiated partnerships with the American Board of Medical Specialties (ABMS), Program Director Associations, and residents to identify and implement specialty specific milestones to further promote outcomes based training.^6^ Several other specialties have highlighted the theoretical basis for, and advantages of, their milestones.^7^^,^^8^ Milestone advantages include enhanced learner feedback, greater uniformity of evaluation, facilitation of remediation, and curricular improvements.^9^ Early pilots confirmed these benefits in programs that implemented milestones.^10^ Seven early adopter specialties, including Orthopaedic Surgery, committed to be the first specialties to implement milestones nationally in all of their ACGME accredited graduate medical education programs beginning in July 2012. Five of these seven, including Orthopaedic Surgery, committed to also assess each of their residents’ performance against milestones twice yearly beginning in December 2013, and report these data to the ACGME.  The remaining specialties have followed in subsequent years.  All residency programs should begin milestone reporting to ACGME, at the latest, in spring and winter of 2016.

ACGME requires each program to use a clinical competency committee (CCC) to judge resident performance along a defined progression of cognitive, professional, and technical milestones.  Milestones represent the progressive trajectory of a resident’s expected skill development from basic to aspirational. Most specialties have identified between 13 and 41 milestones and have provided narrative descriptions of five performance levels along this trajectory.   As a CBE tool, it is important to note that milestones are not meant to correspond to “time” in training.  For example, it may be tempting to think of Level 1 as representing mastery of the first postgraduate year.  However, some learners may be able to reach or exceed Level 1 much more quickly than others. This may be a consequence of a variety of factors, such as additional experiences in medical school prior to residency, or a difference in learning speed.   ACGME predicts that Level 4 is a reasonable potential resident graduation target in most specialties, but has not instituted a graduation requirement at this time. Because the milestones are new, it is not known if all residents will achieve Level 4 on all, some, or even any of the currently published milestones by program completion. The eventual release of ACGME milestone data on all residents will create trajectories of specialty specific aggregate resident performance.  Such milestone data will allow programs to compare how their own residents’ trajectories compare to the trajectory of all residents nationwide. They will allow programs to more easily and confidently identify outliers on both ends of the performance spectrum, who may benefit from curricular enhancements or earlier remediation.

ACGME did not intend for the milestones to be substituted for the program’s current assessment methods.  Regardless, many programs have substituted actual milestone language for their current assessment tools, at least to some extent. For truly accurate assessment of resident proficiency, multiple types of assessment tools and multiple assessors are necessary.  One such assessment tool is “self-assessment”.  ACGME has long recognized resident “self-assessment” as both a tool and as a foundational skill in the competency of Practice Based Learning and Improvement. Because the ability to self-assess has been believed to be essential to physicians’ lifelong learning, ACGME previously required programs to document how they developed their trainees’ abilities’ in self-assessment and reflection. This was a required component of the Common Program Information Form, which was formerly required for program accreditation.^11^^,^^12^ Also, self-assessment is explicitly included as one of the four components of the American Board of Medical Specialties’ Maintenance of Certification (MOC) program: Part 2 Life Long Learning and Self-Assessment.  Virtually all board certified physicians, (93%) are anticipated to be participating in a Maintenance of Certification program by 2020.^13^ However, whether physicians are capable of accurate self-assessment has been a subject of active debate, not just in the US, but also globally.^14^^-^^16^ Methodological controversy is frequent.^17^ Recent experts have posited that the construct of self-assessment has been insufficiently informed by other cognitive disciplines. Rather than a stable skill, self-assessment is a “situationally bounded cognitive process, context specific and dependent upon expertise.” Colthart‘s team reviewed 77 of 5790 published articles on self-assessment from 1990-2006 and concluded “the accuracy of self-assessment can be enhanced by feedback, …explicit criteria, and benchmarking guidance.”^18^^,^^19^

Surgical training may contain many of the elements that might portend “better” self-assessment.  There are often more explicit expectations of performance.^20^^-^^23^ Zevin identified other factors in surgical training that improved self-assessment: higher levels of expertise of the learners, high quality timely and relevant feedback, and the ability for post procedure video review.^24^ Direct observation and frequent formative feedback may help residents calibrate their own impressions with those of their faculty.  Surgical workplace clinical “outcomes” may be observed more directly and immediately related to performance. Reviewing case logs provides continual opportunities for self-reflection, on both the individual’s relative strengths and weaknesses, and as a benchmark for comparison to peers. Mandel et al studied 74 residents’ from 5 institutions. They demonstrated the self-assessment of open and laparoscopic skills was valid and reliable.^25^   Traijkovski demonstrated the self-assessments of competency of 17 Orthopaedic residents performing 65 primary total knee and hip arthroplasty procedures were similar to staff ratings.^26^ Shanedling’s group utilized a self-reported “perception of preparedness” (P of P) measure with 28 Orthopaedic residents and 2 medical students in the context of carpal tunnel release surgery. The P of P is a type of self-assessment.  The P of P was compared to scores from an online 100-item cognitive “medical knowledge” test. The internal consistency of the P of P was better than the cognitive test (α = .92 vs. .65) and more likely to correctly predict subsequent successful carpal tunnel release surgery performance on cadaveric specimens. (76% vs. 73%).^27^

The transition of graduate medical education to CBE using milestones is in an exciting phase. There is tremendous work to be done to integrate milestones into curricula and assessment, faculty development, and the work of clinical competency committees.^28^ Increasingly milestones will be used in undergraduate medical education for curricular activites^29^^,^^30^ and for the transition to graduate medical education.^31^^,^^32^ In some cases they may result in shortened duration of training. Researchers are also beginning to describe institutional and programmatic uses of milestones and how they are informed by various assessment strategies.^33^ Residents attribute improved feedback to the use of milestones.^34^ Programs report benefits in improving their assessments and identifying and remediating their lower performers.^35^^-^^37^ As with any change, not all programs feel optimally prepared.^38^ Logistic and ethical challenges remain for many.^39^

Though not the only tool in determining how resident performance stacks up against milestones, self-assessment will provide an important data element that can be considered in context with others.  It can also help programs provide residents with feedback on their self-assessment abilities, and hopefully help them improve their own self-assessments. Program directors will be able to combine milestone data with their existing assessment tools to benchmark their own residents’ trajectory of skill acquisition.  They will be able to compare the progress of their resident cohorts by training year, and their overall training program to the national experience. As yet, however, ACGME has not released milestone aggregate data for all specialties, so comparisons to national peer groups are not possible.

The objective of this study was to demonstrate if residents could self-assess their performance using milestones. The time frame was deliberately selected to be at the conclusion of the academic year just prior to the adoption of milestones. At this point in time, most programs had not yet devoted much curricular attention to the milestones themselves. Milestones were written to reflect each of ACGME’s six core competencies: Patient Care (PC), Medical Knowledge (MK), Practice Based Learning and Improvement (PBLI), Systems Based Practice (SBP), Professionalism (P) and Interpersonal and Communication Skills (IC). Anecdotally, programs seem to be more confident assessing PC and MK, than the perhaps “softer” skills of PBLI, SBP, P and IC.  In this study the following hypotheses were tested:  1) Orthopaedic residents will be able to self-assess using milestones, 2) Self-assessment scores will increase across postgraduate training years one through five, 3) Self-assessment scores for milestones will differ by competency; specifically, patient care (PC) and medical knowledge (MK) milestones will differ compared with the four general competencies: practice based learning and improvement (PBLI), systems based practice (SBP), professionalism (P), and interpersonal and communication skills (IC).

## Methods

### Study design

This is a cross sectional descriptive study of Orthopaedic residents who completed an online survey in which they self-assessed their competency using narrative descriptions provided by the milestones.  The survey contained forty-five questions. Four questions were demographic variables. Forty-one questions were adapted from the Orthopaedic Surgery Milestone Project, a Joint Initiative of the Accreditation Council for Graduate Medical Education and the American Board of Orthopaedic Surgery, version December 2012.^40^ Expert panels, selected to be representative of the specialty, developed all of the milestones. The survey included sixteen PC milestones (such as Ankle Fracture): sixteen corresponding MK Milestones, and milestones from nine General Competency domains (PBLI, SBP, P, IC).

For each milestone, blank boxes below the descriptions of level 1 to level 5 were labeled A through I. Residents were asked to select the milestone “box” which best characterized their current performance level. The letters were subsequently converted to a numeric scale, 1-9.  Choice A, (corresponding to “1”), indicated the resident believed he or she had substantially achieved the Level 1 milestone.  Choice B, (corresponding to “2”), indicated the resident believed he or she had substantially achieved all Level 1 milestones and some Level 2 milestones.  Choice C, (corresponding to “3”), indicated the resident believed he or she had substantially achieved all Level 2 milestones.  Choice D, (corresponding to “4”), indicated the resident believed he or she had substantially achieved all Level 2 milestones and some Level 3 milestones.  Choice E, (corresponding to “5”), indicated the resident believed he or she had substantially achieved all Level 3 milestones.  Choice F, (corresponding to “6”), indicated the resident believed he or she had substantially achieved all Level 3 milestones and some Level 4 milestones.  Choice G, (corresponding to “7”), indicated the resident believed he or she had substantially achieved all Level 4 milestones. Choice H, (corresponding to “8”), indicated the resident believed he or she had substantially achieved all Level 4 milestones and some Level 5 milestones.  Choice I, (corresponding to “9”), indicated the resident believed he or she had substantially achieved all Level 5 milestones. The study addressed all of the ethical concerns in graduate medical education research identified by Keune.^41^   Ethical approval was obtained by submitting the study plan to our Institutional Review Board, which determined the study exempt from requirements for further review, thereby allowing the study to proceed. Aggregate results were offered to program directors at the end of the study regardless of whether or not they had requested their residents participate.

### Participants

Orthopaedic Surgery residents who voluntarily responded to an email survey forwarded to them by their program director or coordinator were included in the study.

### Sample size and sampling methods

In June 2013, an email was sent to the Program Directors and coordinators of the 154 ACGME accredited Orthopaedic Surgery Programs in which approximately 3501 residents were training. Email addresses were identified from the ACGME public database or other public institutional directories.  The email explained the study’s purpose and asked them to send an email to their residents containing a link to a voluntary survey constructed in RedCAP.^42^

### Data collection

Respondents completed all surveys online. Their responses populated an anonymous, confidential database in REDCap that captured de-identified aggregate information. The data were stored on a secure server.

### Data analysis

SAS 9.3 was used to perform the analyses. A one-way ANOVA was performed to determine if there were differences in how residents self-assessed their abilities across residency years. Two-sample t tests were used to determine if the average of each milestone group was different.  P-values were adjusted using empirical methods to account for multiple comparisons.  Aggregate scores from all of the milestones within each of the PC and MK competencies were compared with aggregate scores from the milestones of the other 4 competencies by postgraduate training years. Two-sample t-tests were used to determine if the averages differed.  P-values were adjusted to account for multiple comparisons.

## Results

Seventy-four residents responded to the study. Seventy-one completed the survey in its entirety and indicated their gender and postgraduate year (PGY) 1 to 5, as there are five postgraduate years in ACGME accredited Orthopaedic residency programs. Responses from those 71 are included in this analysis.  There were 14 PGY1s; 10 PGY2s; 17 PGY3s; 16 PGY4s; and 14 PGY5s. There were no women among the PGY1 respondents.  There were two women PGY2s and PGY3s sand three women among the PGY3s and PGY5s. The self-assessment of PGY1s, (first year residents), and PGY2s, (second year residents), indicated they believed they had substantially achieved Level 1 and Level 2, respectively, for all milestones. PGY3s, (third year residents) reported they had substantially achieved 30/41, and PGY4s (fourth year residents) all Level 3 milestones.  PGY5s (fifth and final year residents) reported they had substantially achieved 17 Level 4 milestones and were “extremely close” to substantially achieving another 15. PGY5 residents only rated two milestones at less than the “midpoint” between Level 3 and 4. Interestingly, no resident scored him or herself as a “9,” the highest possible level and corresponding to substantially achieving Level 5, on any milestone (see Table 1).

**Table 1:** Orthopaedic resident mean self-assessment by postgraduate year (PGY) 1-5 on each orthopaedic surgery milestone

Orthopaedic Surgery Milestone	PG Yr1	PG Yr2	PG Yr3	PG Yr4	PG Yr5
Anterior cruciate ligament-Patient Care	2.00	3.13	4.75	5.81	6.29
Anterior cruciate ligament-Medical Knowledge	2.73	4.43	5.19	5.79	6.50
Ankle Arthritis-Patient Care	1.80	3.44	4.85	5.94	6.86
Ankle Arthritis- Medical Knowledge	1.50	3.78	3.77	5.19	5.93
Ankle Fracture-Patient Care	2.43	4.50	5.76	6.81	7.36
Ankle Fracture-Medical Knowledge	2.50	4.80	5.94	6.50	7.00
Carpel tunnel-Patient Care	2.55	4.50	5.75	7.06	6.71
Carpel tunnel-Medical Knowledge	2.55	4.13	5.56	6.63	6.43
Degenerative Spine Conditions-Patient Care	2.27	3.89	4.88	5.88	6.14
Degenerative Spine Conditions-Medical Knowledge	2.09	3.67	4.35	5.93	5.79
Diabetic Foot-Patient Care	2.08	3.60	4.93	5.25	6.79
Diabetic Foot-Medical Knowledge	2.15	3.60	4.64	5.69	6.50
Diaphyseal Femur and Tibia Fracture-Patient Care	2.50	4.78	5.47	6.56	7.36
Diaphyseal Femur and Tibia Fracture-Medical Knowledge	2.64	5.00	5.76	6.63	6.64
Distal Radius Fracture-Patient Care	2.29	5.00	5.29	7.13	7.57
Distal Radius Fracture-Medical Knowledge	2.71	5.22	5.71	6.63	6.64
Adult Elbow Fracture-Patient Care	1.79	3.78	4.65	5.88	6.71
Adult Elbow Fracture-Medical Knowledge	1.86	3.56	4.71	5.75	6.43
Hip and Knee Osteoarthritis-Patient Care	2.62	4.20	5.53	6.50	7.21
Hip and Knee Osteoarthritis-Medical Knowledge	3.08	4.50	5.82	6.63	7.07
Hip Fracture-Patient Care	2.64	4.80	5.71	6.25	7.57
Hip Fracture-Medical Knowledge	2.71	4.80	5.71	6.25	7.21
Metastatic Bone Lesion-Patient Care	1.67	3.50	4.38	5.63	6.29
Metastatic Bone Lesion-Medical Knowledge	1.78	4.00	4.23	5.38	6.29
Meniscal Tear-Patient Care	2.09	4.38	5.20	5.75	6.57
Meniscal Tear-Medical Knowledge	2.18	4.00	5.67	6.07	6.62
Pediatric Septic Hip-Patient Care	2.36	4.44	5.00	6.44	6.57
Pediatric Septic Hip-Medical Knowledge	2.09	4.56	5.00	6.19	6.43
Rotator Cuff Injury-Patient Care	1.91	3.75	5.13	5.88	6.64
Rotator Cuff Injury-Medical Knowledge	2.18	3.75	5.67	6.44	7.14
Pediatric Supracondylar Humerus Fracture-Patient Care	1.91	4.33	5.63	6.50	7.00
Pediatric Supracondylar Humerus Fracture-Medical Knowledge	1.91	4.33	5.56	6.31	6.57
Systems Thinking-Systems Based Practice	2.85	4.60	5.75	6.20	6.86
Interprofessional Teams, patient safety/ quality-Systems Based Practice	3.46	4.50	5.69	6.19	6.57
Technology-Systems Based Practice	4.62	4.60	5.69	6.94	7.08
Self-directed learning-Practice Based Learning & Improvement	3.86	5.00	6.13	6.25	7.36
Locate, appraise and assimilate evidence-Practice Based Learning and Improvement	3.57	4.50	5.88	5.81	7.07
Compassion, integrity respect-Professionalism	4.29	6.10	6.50	7.38	7.31
Accountability to patients, society and the profession-Professionalism	4.07	5.30	6.06	6.94	7.21
Communication-Interpersonal and Communication Skills	4.38	5.78	6.06	6.88	7.21
Teamwork-Interpersonal and Communication Skills	4.15	5.50	6.00	6.56	7.21

**Figure 1:**
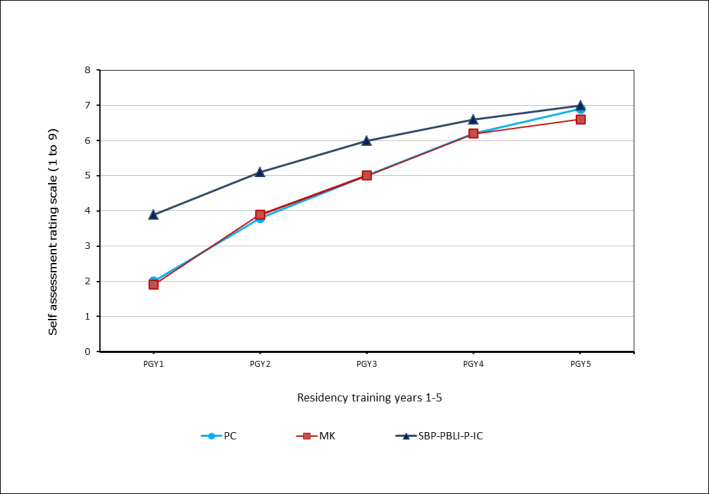
Trajectory of aggregate milestone acquisition combined for competencies of Patient Care (PC), Medical Knowledge (MK), and pooled Systems Based Practice (SBP)-Practice Based Learning and Improvement (PBLI)-Professionalism (P)-Interpersonal and Communication (IC) by postgraduate years 1-5

Of the 16 paired PC and MK milestones, PGY1s self-assessed MK more highly than PC for 11 milestones, and “the same” for two milestones.  Their lowest rated milestone was MK-ankle arthritis and their highest rated milestone was the SBP milestone, “use of technology.”  PGY1s reported some achievement of Level 2 on twenty-three milestones; substantial achievement of level 2 on four milestones; and partial achievement of Level 3 on five milestones.  Eight of the nine most highly rated milestones were in the four general competencies of PBLI, SBP, P and IC.  Mean PGY2 self-assessments were higher than PGY1 self-assessments for forty of forty-one milestones. The average self-assessment of technology skills was essentially the same: 4.62 for PGY1 and 4.60 for PGY2s.  There were statistical differences for 15 of 16 PC milestones, 13 of 16 MK Milestones and 2 of 9 PBLI-SBP-P-IC milestones. Aggregate PC, MK, and PBLI-SBP-P-IC milestones were also statistically different from PGY1 to PGY 2. Figure 1 presents the trajectory of milestone acquisition for the aggregate of PC, MK and the general competency domains of SBP, PBLI, P, and IC by PGY 1-5. The Y-axis displays the rating scale, 1-9, used by residents in their self-assessment. The X-axis represents residents at the conclusion of each of the five postgraduate residency training years 1-5.

**Figure 2:**
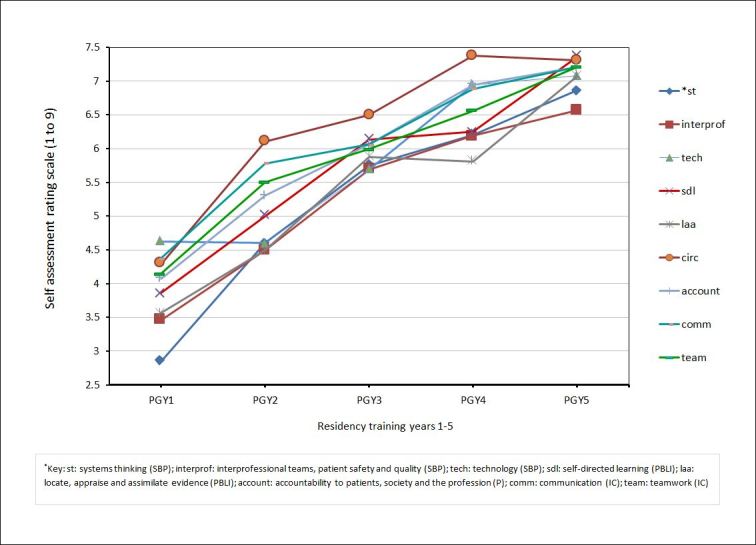
Trajectory of milestone acquisition by postgraduate year for each of the general competency domains of Systems Based Practice (SBP), Practice Based Learning and Improvement (PBLI), Professionalism (P), and Inter-personal and Communication Skills (IC) by postgraduate years 1-5. *st: systems thinking (SBP); interprof: Interprofessional teams, patient safety and quality (SBP); tech: technology (SBP); sdl: self-directed learning (PBLI); laa: locate, appraise and assimilate evidence (PBLI); account: accountability to patients, society and the profession (P); comm: communication (IC); team: teamwork (IC)

Milestones from MK and PC competencies were generally rated less highly than the four general competency milestones.  This difference was statistically different only for PGY1s. See Figure 1. The self -assessed trajectory of skill acquisition varied among the 9 general competency milestones (PBLI, SBP, P, IC). Figure 2 presents the trajectory of milestone acquisition for each of the general competency domains of SBP, PBLI, P, and IC.  The Y-axis is the rating scale, 1-9, used by residents in their self-assessment.  The X-axis represents residents at the conclusion of each of the five postgraduate residency training years 1-5.

## Discussion

We are unaware of any previously published “baseline” self-assessment of residents using milestones prior to their adoption by the first seven specialties in July 2013 and subsequently by the remaining specialties.  A single study of entering Emergency Medicine interns in summer 2012 reported that many of them were unaware of either teaching or assessment on milestones.^43^ While the interns were unaware of the use of milestones, practicing emergency medicine physicians were able to use milestones to self-assess their own performance.^44^

In our pilot study, we found Orthopaedic residents were able to self-assess themselves using the Orthopaedic milestones. They appeared to discriminate among the milestones and did not assess themselves as equally proficient in all forty-one milestones. At the conclusion of the first postgraduate year, first year residents generally felt they “knew” more than they could “do.”  Self-assessment of MK milestones was generally higher than PC milestones. Self-assessment scores increased from the first to the fifth postgraduate year. In general, earlier in training, residents self-assessed themselves as more capable in the PBLI-SBP-P-IC domains, than the MK and PC domains. This gap essentially closed by the time they were PGY4s. The trajectory of skill acquisition also varied depending on which milestone was assessed.

We believe there is still more to be learned about self-assessment, including how to best correlate it with other sources of assessment. One hundred and eighty-one internal medicine resident self-assessments of their religious/spiritual communication competency correlated well with 541 patients’ ratings of their communication.^45^ Aggregated self-assessment may also play an important role in evaluating program effectiveness, in addition to measuring resident performance, self-assessment may outperform end of rotation evaluations as a measure of program quality.^46^

The study was not designed to compare resident assessment with the assessments of their faculty.  We have no comparison faculty or program ratings and therefore we cannot address whether residents overinflated or underinflated their achievement.  However, at least by one measure, residents seemed not to overinflate their proficiency.   No resident indicated achievement of Level 5, which was the highest level possible.  None self-assessed their performance even at a level midway between Level 4 and Level 5 by the time of program completion. This may be in keeping with findings by Gow and others, who indicate more advanced learners, are better at self-assessment and more critical of their own performance than even their attendings.^47^ It is also possible they actually underestimated their performance. Our respondents self-assessed their own abilities to self-assess very highly.  PGY1s ranked the milestone of “self-directed learning”, which includes the ability to “accurately assess (their) areas of competency and deficiencies,” as their sixth most highly rated milestone. This milestone was tied for the sixth most highly rated milestone for PGY2s.   PGY3s rated it as the second highest. By the completion of PGY2, residents’ self-assess their self-assessment skills as Level 3. Future work should compare resident milestone self-assessment with other assessment tools, and the program’s clinical competency committee’s judgment of their performance. 

The general competency milestones (PBLI-SBP-P-IC) were rated more highly than PC or MK milestones, though this was statistically significant only at the PGY1 level.  This is consistent with Blanch-Hartigan’s findings that medical students, as a whole, do not over or underestimate their skills, but are more likely to overestimate their performance on communication skills than knowledge.^48^

If first year residents overestimated their skills, it may have been because the lowest option possible corresponded to “substantially achieved Level 1.”  Unlike some other specialties’ milestones, there is no “column to the left” of Level 1, indicating the resident “has not yet substantially achieved Level I” (as do the Emergency Medicine milestones) or, has a “critical deficiency” (as do the Internal Medicine milestones).  Orthopaedic Surgery may wish to consider adding such an option to help better distinguish performance among the more junior residents. A simple change would be to include the option of “not yet achieved Level 1” for PC and MK Milestones, not just the PLBI-SBP-P-IC milestones.

We note with interest that the PBLI-SBP-P-IC milestones were rated more highly than PC and MK milestones. By PGY4, the gap between PBLI-P-SBP-P-IC milestones’ and the MK and PC milestones had substantially been erased. But residents also expressed variability in the PBLI-SBP-P-IC milestones’ developmental trajectory. Those also differed by milestone.

Interestingly some of the milestones PGY1s rated the highest, such as technology, were less highly rated by PGY2s and PGY3s.  Perhaps they became better aware of their true skill level as they matured, or the nature of their work in those years required awareness of a skill level beyond their original conception of proficiency.  In other words, they learned that there was still more to be learned. 

It is anticipated that milestones will benefit other types of assessment. Faculty assessment of resident performance may be improved. Milestones use narrative descriptions of performance, and such narrative descriptions have outperformed the use of numeric scales in helping faculty more authentically assess residents.^49^ Narrative descriptions of performance may help residents better gauge and calibrate their own performance to program expectations.^50^ They may help faculty and residents develop a shared mental model regarding performance expectations. This could facilitate the educational alliance described by Telio^51^ that is so essential in improving the feedback process.

Our pilot study confirmed Level 4 milestones represented a realistic target for this sample of residents. PGY5 respondents, on average, reported they had substantially achieved 17 Level 4 milestones and were extremely “close” to achieving another 15.  They reported they had substantially achieved competency in Level 3 with some competency in Level 4 for another 7 milestones. There were only two milestones on which they “rated” themselves lower than the midpoint between Level 3 and 4. Both of these were MK milestones: ankle arthritis and degenerative spinal conditions.  Interestingly, Orthopaedic residents have expressed their belief in the importance of case logs in demonstrating performance in these two clinical areas.^52^ PGY1 self-assessment did not predict the assessment at PGY5. PGY1s ranked ankle fracture-pc and ankle arthritis-mk as their two lowest milestones. Only ankle arthritis-pc remained as one of the two lowest rated milestones ranked by the PGY5s. PGY5s ranked distal radius facture-pc and degenerative spinal conditions-mk as the most highly rated milestones.  PGY1s ranked distal radius fracture-pc fourteenth of the thirty-two PC-MK milestones, and degenerative spinal conditions-mk tied for 18th.

There are number of limitations to our study.  We are well aware that our 71 participants represent only a small proportion of the country’s Orthopaedic residents.  Our study design utilized a single email request to Program Directors and residency coordinators. We were concerned about survey fatigue and aware that June is an extremely busy time in residencies.  However because we wanted to “biopsy” residents at the conclusion of their training year, when presumably their competence would be the highest, and just prior to milestone implementation, June was the most appropriate month.  We suspect the response rate would have been higher if we had emailed residents directly, or followed up on our initial request with additional emails.  We also do not know either how many, or which programs, are represented in our respondents.  The number of respondents confirms that more than a single program is represented in the study, since no single program has over 70 residents. Our results may be biased if our sample resulted in residents who would rate themselves disproportionately to their colleagues. We note we have few women respondents and the self-assessments of women may differ from their male colleagues.  Orthopaedic Surgery is the US specialty with the fewest percentage of women residents.  Our survey appeared to be representative as 14% of our sample were women, compared with 13% of women Orthopaedic residents nationally.^53^

As a cross sectional study, we did not follow the same cohort of residents across five years of residency, but rather “biopsied” residents from each training year, at a single point in time, at the conclusion of an academic year. The mean values overall increased from PGY1-5. But we acknowledge we may have missed individual variability and some individuals’ self-assessment of proficiency may have plateaued or even declined. We have no way of knowing how the respondents’ own programs would have rated their milestone acquisition. Nor are we able to ascertain how their self-assessment compared with any other measure of their performance, such as their in-service examination scores, direct observation by faculty, or multisource feedback from nurses and peers.  We believe the anonymity of the survey counteracted some recognized concerns of self-assessments, such as when learners are concerned their own low self-assessment will result in a lower or negative evaluation from their program.

The two milestones assessed as the weakest at program completion may not be representative if there were a larger sample of residents reporting.  All Orthopaedic programs submitted their graduating residents’ performance on milestones to ACGME beginning in June 2013. However, ACGME has not yet publicly released those aggregate milestone reports. When released, we will be interested in comparing the milestones that nationally are felt to be relatively stronger and weaker with our findings. Those milestones on which there is relatively weaker performance will provide rich opportunities for curricular development and resident coaching within individual programs.  It will allow the specialty to engage in conversations about realistic targets, training opportunities, and its own scope of practice.

The milestones we presented to the residents in this study were those posted on the ACGME web site as of May 2012.  There is currently a posted version that is dated August 2013.^54^ A comparison of the two documents indicates fairly minimal editorial changes. We do not believe the differences are sufficient to result in different responses had the residents been presented with the current version.

## Conclusions

Although a limited response, we believe the results from our pilot study support the proof of concept that Orthopaedic Surgery residents can self-assess their performance using milestones. Residents discriminated among the milestones and did not assess they were equally proficient in all.  Respondents report improved abilities across their five training years, but none believed they were at Level 5, the maximum possible. Graduating residents rated themselves substantially achieving, or very close to substantially achieving, Level 4, which ACGME predicts is the likely graduation target.   In aggregate, PBLI-SBP-P-IC milestones were rated more highly than PC or MK milestones. Resident self-assessment using milestones may be a useful assessment tool for programs and CCCs as they assess resident performance. Milestones may facilitate critical conversations between residents and faculty. They may help residents consider other sources of feedback to better calibrate their own self-assessment.  Milestones will provide opportunities for self-directed learning, ultimately supporting habits of life-long, deliberate practice.

### Acknowledgements

Source of Funding: This project was funded internally through a faculty research account that paid for survey construction and database in REDCap.  We appreciate the statistical support partially funded by the National Center for Advancing Translational Science of the National Institutes of Health through Grant Number UL1RR024128.

### Conflict of Interest

The authors declare that they have no conflict of interest.
